# Editorial: Perspectives in brain-network dynamics in computational psychiatry

**DOI:** 10.3389/fncom.2023.1290089

**Published:** 2023-09-22

**Authors:** Sou Nobukawa, Tetsuya Takahashi

**Affiliations:** ^1^Graduate School of Information and Computer Science, Chiba Institute of Technology, Narashino, Japan; ^2^Department of Computer Science, Chiba Institute of Technology, Narashino, Japan; ^3^Research Centre for Mathematical Engineering, Chiba Institute of Technology, Narashino, Japan; ^4^Department of Preventive Intervention for Psychiatric Disorders, National Institute of Medicine Mental Health, National Center of Neurology and Psychiatry, Tokyo, Japan; ^5^Research Centre for Child Mental Development, Kanazawa University, Kanazawa, Japan; ^6^Department of Neuropsychiatry, Faculty of Medical Sciences, University of Fukui, Yoshida, Japan; ^7^Uozu Shinkei Sanatorium, Uozu, Japan

**Keywords:** models, network dynamics, neural circuits, multi-temporal scale, neuroimaging, psychiatry

## 1. Introduction

The cognitive functions of the brain are achieved through mutual interactions between various hierarchical regional neural activities (reviewed in Sporns, 2016). Throughout the current decade, studies on brain networks have revealed the dynamical behaviors of brain networks, including multitemporal scale dynamics, from ultra-slow to moment-to-moment behaviors (Ando et al., 2021, 2022; Iinuma et al., 2022; Gandhi et al.) (reviewed in Garrett et al., 2013; Takahashi, 2013; Palva and Palva, 2018). In these multiscale neural activities, the network dynamics, which is captured by the degree of synchronization and information flow between pair-wise brain regional neural activities [called dynamic functional connectivity (dFC)], play an important role in coordinating the mutual interactions of neural activities (reviewed in Cohen, 2018; Luppi et al., 2022). Moreover, besides the pair-wise neural interactions, the temporal itinerancy of the global topology of the whole-brain functional network is present (Guan et al., 2022). Under pathological conditions, the multitemporal scale characteristics of such network dynamics exhibit disease-specific alternations (Yan et al., 2023). These characteristics therefore pose the possibility of realizing potential biomarkers to identify psychiatric disorders. For achieving this, we have two major approaches.

The first is a physiological data-driven neuroimaging approach using electroencephalography (EEG), magnetoencephalography (MEG), and functional magnetic resonance imaging (fMRI). For this approach, the method for utilizing the temporal variation of functional connectivity within a short time window has been developed (Damaraju et al., 2014). Subsequently, a more rigid method for determining the length of a window based on the temporal transition of a quasistable spatial power distribution, called a microstate, was proposed (Guan et al., 2022; Yan et al., 2023). Moreover, instead of focusing on synchronization within the time-window, a technique of utilizing the instantaneous temporal patterns produced by neural interaction was also developed, which is required for achieving high temporal resolution to capture the characteristics of moment-to-moment dynamical functional connectivity (Nobukawa et al., 2019).

The second approach is a simulation-based one using mathematical models with high pathological validity, typified as abstract whole-brain neural networks and spiking neural networks (reviewed in Cabral et al., 2017; Nobukawa, 2022). Recent mathematical modeling of brain networks focuses on large hierarchical neural characteristics from the molecular/cellular and local neural circuit levels to the global whole brain level. Therefore, embedding disease-specific impairments into the modeled-brain network studies could reveal the mechanisms by which these individual impairments affect the alternations of brain network dynamics (Matsumoto et al., 2023; Park et al.; Zhu et al.).

This Research Topic is intended to inspire further research focusing on both approaches, and to facilitate the mutual use of the findings of network dynamics and individual approaches. This editorial briefly explains the studies based on these approaches.

## 2. Network dynamics in the physiological-data approach

Studies in this decade have revealed that functional connectivity exhibits large temporal variability, even in the resting state, which is called dFC (Betzel et al., 2012; Hutchison et al., 2013; Allen et al., 2014; Calhoun et al., 2014; Hansen et al., 2015) (reviewed in Cohen, 2018). These network dynamics possess quasistable states, temporal transitions, and hierarchical sequential characteristics (Vidaurre et al., 2017), instead of random characteristics. These dynamical characteristics strongly relate to cognitive functions [e.g., executive functions (Braun et al., 2015), associative learning (Bassett et al., 2011), perceptions (Frolov et al., 2019), and deficits in cognitive functions in various pathological conditions such as, schizophrenia (Damaraju et al., 2014), autism (Guo et al., 2020), and Alzheimer's disease (Gu et al., 2020)] (reviewed in Gonzalez-Castillo and Bandettini, 2018; Sporns, 2022). In this Research Topic, Gandhi et al. specifically evaluated neurophysiological differentiation as a measure of dynamical network states related to the subjective perception of visual stimuli. Notably, despite the use of neuropixels recordings as an invasive method, these findings could potentially aid in the identification of biomarkers for psychiatric disorders characterized by dysfunctional perceptual processes.

## 3. Network dynamics in the simulation-based approach

In addition to the experimental studies based on physiological data, the simulation-based approach with mathematical modeling is effective for revealing factors that induce the alteration of network dynamics by embedding the network structural characteristics into the neural networks (Lea-Carnall et al.; Barkdoll et al.; Park et al.; Zhu et al.) (reviewed in D'Angelo and Jirsa, 2022). Particularly, in this Research Topic, Lea-Carnall et al. demonstrated that dynamical network patterns appear according to the fluctuation level, which is suggestive of dFC. Furthermore, it is widely recognized that an imbalance of excitatory and inhibitory neural activity leads to abnormal neural activity observed under pathological conditions and deficits in cognitive function (reviewed in Yizhar et al., 2011; Bosman et al., 2014). Park et al. reported that a locally increased excitatory/inhibitory ratio prevents information flow in neural networks; subsequently, the complexity as the degree of mutual interactions among neural populations reduces, and Zhu et al. further demonstrated that the deficits of top-down control and inhibitory effects lead to a schizophrenia-like illusion representation. Barkdoll et al. constructed a neural network model for the emergence of binocular rivalry and showed that impairment of inhibitory neural activity leads to the slow percept switching in binocular rivalry, which resembles the tendency observed in autism.

## 4. Conclusions

In this editorial, we have explained several recent studies, particularly including those acquired studies in this Research Topic (Lea-Carnall et al.; Gandhi et al.; Barkdoll et al.; Park et al.; Zhu et al.), for network dynamics using physiological-data and simulation-based approaches. We believe that the findings of these studies will interact to facilitate future biomarker development for psychiatric disorders.

## Author contributions

SN: Writing—original draft, Writing—review and editing. TT: Writing—original draft, Writing—review and editing.

## References

[B1] Allen E. A. Damaraju E. Plis S. M. Erhardt E. B. Eichele T. Calhoun V. D. (2014). Tracking whole-brain connectivity dynamics in the resting state. Cereb. Cortex 24, 663–676. 10.1093/cercor/bhs352 23146964 PMC3920766

[B2] Ando M. Nobukawa S. Kikuchi M. Takahashi T. (2021). Identification of electroencephalogram signals in Alzheimer's disease by multifractal and multiscale entropy analysis. Front. Neurosci. 772, 667614. 10.3389/fnins.2021.667614 34262427 PMC8273283

[B3] Ando M. Nobukawa S. Kikuchi M. Takahashi T. (2022). Alteration of neural network activity with aging focusing on temporal complexity and functional connectivity within electroencephalography. Front. Aging Neurosci. 14, 793298. 10.3389/fnagi.2022.793298 35185527 PMC8855040

[B4] Bassett D. S. Wymbs N. F. Porter M. A. Mucha P. J. Carlson J. M. Grafton S. T. (2011). Dynamic reconfiguration of human brain networks during learning. Proc. Nat. Acad. Sci. U. S. A. 108, 7641–7646. 10.1073/pnas.1018985108 21502525 PMC3088578

[B5] Betzel R. F. Erickson M. A. Abell M. O'Donnell B. F. Hetrick W. P. Sporns O. (2012). Synchronization dynamics and evidence for a repertoire of network states in resting EEG. Front. Comp. Neurosci. 6, 74. 10.3389/fncom.2012.00074 23060785 PMC3460532

[B6] Bosman C. A. Lansink C. S. Pennartz C. M. (2014). Functions of gamma-band synchronization in cognition: from single circuits to functional diversity across cortical and subcortical systems. Eur. J. Neurosci. 39, 1982–1999. 10.1111/ejn.12606 24809619

[B7] Braun U. Schäfer A. Walter H. Erk S. Romanczuk-Seiferth N. Haddad L.. (2015). Dynamic reconfiguration of frontal brain networks during executive cognition in humans. Proc. Nat. Acad. Sci. U. S. A. 112, 11678–11683. 10.1073/pnas.1422487112 26324898 PMC4577153

[B8] Cabral J. Kringelbach M. L. Deco G. (2017). Functional connectivity dynamically evolves on multiple time-scales over a static structural connectome: models and mechanisms. Neuroimage 160, 84–96. 10.1016/j.neuroimage.2017.03.045 28343985

[B9] Calhoun V. D. Miller R. Pearlson G. Adalı T. (2014). The chronnectome: time-varying connectivity networks as the next frontier in fMRI data discovery. Neuron 84, 262–274. 10.1016/j.neuron.2014.10.015 25374354 PMC4372723

[B10] Cohen J. R. (2018). The behavioral and cognitive relevance of time-varying, dynamic changes in functional connectivity. Neuroimage 180, 515–525. 10.1016/j.neuroimage.2017.09.036 28942061 PMC6056319

[B11] Damaraju E. Allen E. A. Belger A. Ford J. M. McEwen S. Mathalon D.. (2014). Dynamic functional connectivity analysis reveals transient states of dysconnectivity in schizophrenia. NeuroImage Clin. 5, 298–308. 10.1016/j.nicl.2014.07.003 25161896 PMC4141977

[B12] D'Angelo E. Jirsa V. (2022). The quest for multiscale brain modeling. Trends Neurosci. 45, 777–790. 10.1016/j.tins.2022.06.007 35906100

[B13] Frolov N. S. Maksimenko V. A. Khramova M. V. Pisarchik A. N. Hramov A. E. (2019). Dynamics of functional connectivity in multilayer cortical brain network during sensory information processing. Eur. Phys. J. 228, 2381–2389. 10.1140/epjst/e2019-900077-7

[B14] Garrett D. D. Samanez-Larkin G. R. MacDonald S. W. Lindenberger U. McIntosh A. R. Grady C. L. (2013). Moment-to-moment brain signal variability: a next frontier in human brain mapping? Neurosci. Biobehav. Rev. 37, 610–624. 10.1016/j.neubiorev.2013.02.015 23458776 PMC3732213

[B15] Gonzalez-Castillo J. Bandettini P. A. (2018). Task-based dynamic functional connectivity: recent findings and open questions. Neuroimage 180, 526–533. 10.1016/j.neuroimage.2017.08.006 28780401 PMC5797523

[B16] Gu Y. Lin Y. Huang L. Ma J. Zhang J. Xiao Y.. (2020). Abnormal dynamic functional connectivity in Alzheimer's disease. CNS Neurosci. Ther. 26, 962–971. 10.1111/cns.13387 32378335 PMC7415210

[B17] Guan K. Zhang Z. Chai X. Tian Z. Liu T. Niu H. (2022). Eeg based dynamic functional connectivity analysis in mental workload tasks with different types of information. IEEE Transact. Neural Syst. Rehabil. Eng. 30, 632–642. 10.1109/TNSRE.2022.3156546 35239485

[B18] Guo X. Duan X. Chen H. He C. Xiao J. Han S.. (2020). Altered inter-and intrahemispheric functional connectivity dynamics in autistic children. Hum. Brain Mapp. 41, 419–428. 10.1002/hbm.24812 31600014 PMC7268059

[B19] Hansen E. C. Battaglia D. Spiegler A. Deco G. Jirsa V. K. (2015). Functional connectivity dynamics: modeling the switching behavior of the resting state. Neuroimage 105, 525–535. 10.1016/j.neuroimage.2014.11.001 25462790

[B20] Hutchison R. M. Womelsdorf T. Allen E. A. Bandettini P. A. Calhoun V. D. Corbetta M.. (2013). Dynamic functional connectivity: promise, issues, and interpretations. Neuroimage 80, 360–378. 10.1016/j.neuroimage.2013.05.079 23707587 PMC3807588

[B21] Iinuma Y. Nobukawa S. Mizukami K. Kawaguchi M. Higashima M. Tanaka Y.. (2022). Enhanced temporal complexity of EEG signals in older individuals with high cognitive functions. Front. Neurosci. 16, 878495. 10.3389/fnins.2022.878495 36213750 PMC9533123

[B22] Luppi A. I. Cabral J. Cofre R. Destexhe A. Deco G. Kringelbach M. L. (2022). Dynamical models to evaluate structure-function relationships in network neuroscience. Nat. Rev. Neurosci. 23, 767–768. 10.1038/s41583-022-00646-w 36207502

[B23] Matsumoto I. Nobukawa S. Kurikawa T. Wagatsuma N. Sakemi Y. Kanamaru T.. (2023). “Optimal excitatory and inhibitory balance for high learning performance in spiking neural networks with long-tailed synaptic weight distributions,” in International Joint Conference on Neural Networks (IJCNN) (Gold Coast, QLD).

[B24] Nobukawa S. (2022). Long-tailed characteristics of neural activity induced by structural network properties. Front. Appl. Math. Stat. 8, 905807. 10.3389/fams.2022.90580727534393

[B25] Nobukawa S. Kikuchi M. Takahashi T. (2019). Changes in functional connectivity dynamics with aging: a dynamical phase synchronization approach. Neuroimage 188, 357–368. 10.1016/j.neuroimage.2018.12.008 30529509

[B26] Palva S. Palva J. M. (2018). Roles of brain criticality and multiscale oscillations in temporal predictions for sensorimotor processing. Trends Neurosci. 41, 729–743. 10.1016/j.tins.2018.08.008 30274607

[B27] Sporns O. (2016). Networks of the Brain (Cambridge, MA: MIT Press).

[B28] Sporns O. (2022). The complex brain: connectivity, dynamics, information. Trends Cogn. Sci. 26, 1066–1067. 10.1016/j.tics.2022.08.002 36207260

[B29] Takahashi T. (2013). Complexity of spontaneous brain activity in mental disorders. Progr. Neuropsychopharmacol. Biol. Psychiatry 45, 258–266. 10.1016/j.pnpbp.2012.05.001 22579532

[B30] Vidaurre D. Smith S. M. Woolrich M. W. (2017). Brain network dynamics are hierarchically organized in time. Proc. Nat. Acad. Sci. U. S. A. 114, 12827–12832. 10.1073/pnas.1705120114 29087305 PMC5715736

[B31] Yan T. Wang G. Liu T. Li G. Wang C. Funahashi S.. (2023). Effects of microstate dynamic brain network disruption in different stages of schizophrenia. IEEE Trans. Neural. Syst. Rehabil. Eng. 31, 2688–2697. 10.1109/TNSRE.2023.3283708 37285242

[B32] Yizhar O. Fenno L. E. Prigge M. Schneider F. Davidson T. J. O'shea D. J.. (2011). Neocortical excitation/inhibition balance in information processing and social dysfunction. Nature 477, 171–178. 10.1038/nature10360 21796121 PMC4155501

